# Femoral head osteochondral allograft transplantation with and without simultaneous periacetabular osteotomy: a case series

**DOI:** 10.1093/jhps/hnaf037

**Published:** 2025-08-04

**Authors:** Sarah J Wegman, Hashim Shaikh, James D Brodell Jr, P Christopher Cook, Brian D Giordano

**Affiliations:** Department of Orthopaedics and Physical Performance, University of Rochester Medical Center, 601 Elmwood Ave, Box 665, Rochester, NY 14620, United States; Department of Orthopaedics and Physical Performance, University of Rochester Medical Center, 601 Elmwood Ave, Box 665, Rochester, NY 14620, United States; Department of Orthopaedics and Physical Performance, University of Rochester Medical Center, 601 Elmwood Ave, Box 665, Rochester, NY 14620, United States; Department of Orthopaedics and Physical Performance, University of Rochester Medical Center, 601 Elmwood Ave, Box 665, Rochester, NY 14620, United States; Department of Orthopaedics and Physical Performance, University of Rochester Medical Center, 601 Elmwood Ave, Box 665, Rochester, NY 14620, United States

## Abstract

Focal femoral head degeneration can lead to loss of structural support of the femoral head and eventual collapse. Formerly, total hip arthroplasty (THA) was the only definitive means available to restore function and mitigate pain. Osteochondral allograft transplantation (OATS) of the femoral head can be used to potentially prevent or delay THA, and the aim of this study is to analyse outcomes of patients who have undergone OATS procedures. An OATS procedure involves vessel-sparing hip dislocation to achieve global femoral head exposure, removal of damaged cartilage and subchondral bone, and press-fit implantation of a fresh femoral head allograft. Eleven patients have undergone OATS at our institution, and we collected retrospective data and Patient-Reported Outcome Measurement Information System (PROMIS) data. All eleven patients demonstrated focal femoral head degeneration. Six patients underwent simultaneous PAO for concomitant structural instability. Postoperatively, patients returned to weight bearing by an average of 9.8 ± 5.6 weeks and physical activities in 9.9 ± 2.9 months. Four patients had unrelated health conditions limiting physical activity involvement. One patient had undergone THA, 6 years postoperatively. Five patients provided updated follow-up metrics, demonstrating significant improvement in average physical function PROMIS scores (*P* = .01). Patients in our series demonstrated sustained favourable outcomes for pain reduction and functional gain at the time of their final follow up. The femoral head OATS procedure may be a reliable treatment with potential to delay THA. In patients with concomitant dysplasia, combined OATS and PAO may provide further enhancement and durability of surgical outcomes.

## INTRODUCTION

Degenerative processes in the femoral head, such as avascular necrosis (AVN) and osteoarthritis (OA), can ultimately lead to structural collapse of the femoral head, resulting in progressive pain and loss of function [1–5]. AVN results from compromised blood supply to focal or diffuse subchondral bone regions [6, 7]. In some instances, AVN is present without clinical symptoms, and in other cases, the individual may experience considerable pain and limited range of motion. With the progression of AVN, diffuse degenerative involvement of the acetabulum eventually occurs [7, 8]. Risk factors for AVN include sickle cell disease, corticosteroid use, trauma, and alcohol abuse [6–12]. OA, on the other hand, occurs due to gradual mechanical damage to the hip joint, resulting in cartilage breakdown, degradative synovial proliferation, and growth of bony prominences [13–15]. OA is often associated with significant pain and limited mobility [16–19]. Risk factors include obesity, muscle weakness, trauma to the joint, and anatomical variation such as joint malignment or femoroacetabular impingement [14, 16, 20].

Both AVN and OA can lead to focal defects of the femoral head that can cause pain, limit function, and progress in severity. In some cases of degeneration, concomitant acetabular dysplasia may influence the force distribution across the joint, both contributing to the underlying pathobiology of the condition and jeopardizing healing and long-term outcomes of surgical reconstruction [21]. Conservative treatments such as restricted weight-bearing and pharmacological agents are often attempted early on in the disease course, but the effectiveness of these treatments is limited [8, 22, 23]. Surgical treatments include core decompression, trap door subchondral decompression, osteotomy, and bone grafting [9, 22, 23], but if the defect progresses to collapse of the femoral head, total hip arthroplasty (THA) is often the only reliable salvage treatment [6, 24, 25]. While THA restores function, certain restrictions are often enforced, and if THA is performed at a young age, the patient may require one or multiple revisions throughout their lifetime [21, 26, 27].

Osteochondral allograft transplantation (OATS) of the femoral head was developed as a method of restoring the structural integrity of both subchondral bone and articular cartilage in cases of focal femoral head degeneration. It allows for preservation of surrounding hip anatomy, which offers the potential for young and active patients to maintain preoperative levels of function and delay the need for THA [28]. Data examining OATS outcomes is sparse, so the purpose of this study is to build upon the limited body of knowledge using our institution’s cohort of OATS patients. Several of these patients presented with concomitant acetabular hip dysplasia requiring simultaneous periacetabular osteotomy (PAO) and femoral head OATS, the outcomes of which have not previously been described [28].

## METHODS

### Ethical approval and patient selection

**Figure 1:**
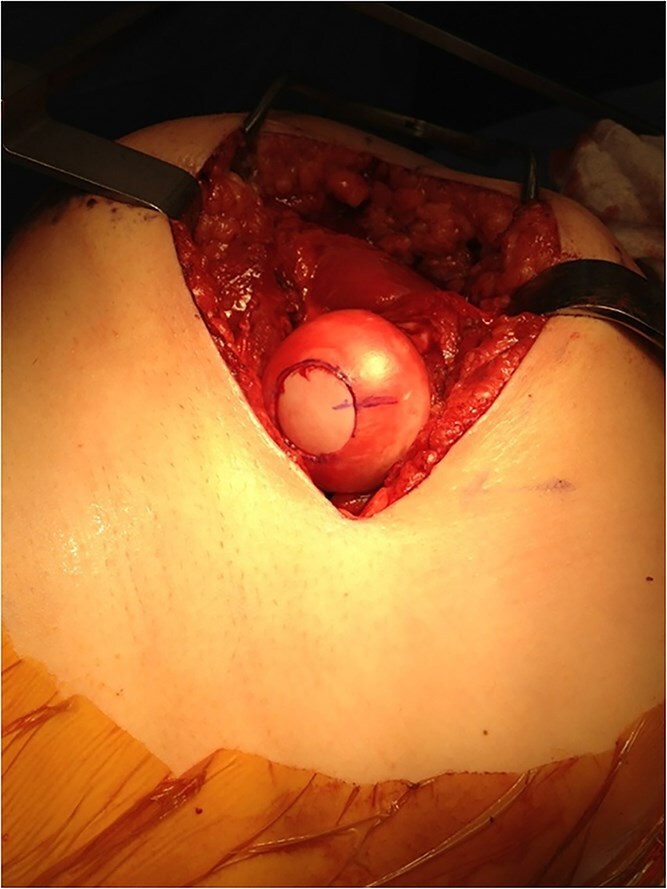
Intra-operative photograph demonstrating femoral head after an OATS procedure.

This study was approved by our institution’s Research Subjects Review Board. We identified 11 patients who underwent OATS procedure at our institution based on the senior author’s case logs. All patients were diagnosed with MRI-confirmed focal femoral head degeneration (Appendix 1). The only inclusion criteria for this study was history of an OATS procedure or both OATS and PAO at our institution, and there were no exclusion criteria used for this study (Fig. 1). The decision to perform PAO was made on a case-by-case basis intraoperatively after analysis of native hip anatomy.

### Data collection

The following data were collected from each patient’s chart: sex, age at time of procedure, Body Mass Index (BMI), risk factors for femoral head degeneration, graft size, surgical history, laterality, Lateral-Center Edge Angle (LCEA), acetabular index, time to weight bearing and/or return to sports, preoperative and >3 months postoperative range of motion, and Patient-Reported Outcome Measurement Information System (PROMIS) data if available. Each patient was contacted *via* telephone, requesting updated physical function, depression, and pain interference PROMIS data. Five patients agreed to participate, one patient declined, five were unable to be reached, and unfortunately, the remaining patient was deceased at the time of follow-up due to unrelated health complications. Each of the five patients gave informed verbal consent after the study information was read to them, and this was documented in compliance with Research Subjects Review Board regulations. They then provided updated PROMIS data *via* surveys over the phone. Final follow-up occurred between 1 and 11 years, with an average of 6.5 years.

**Figure 2:**
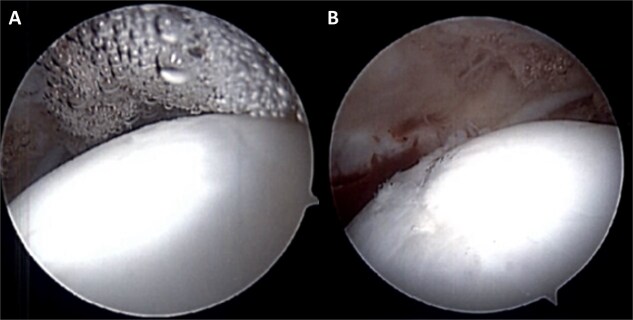
Intra-operative photographs demonstrating excellent graft incorporation postoperatively in two patients who underwent post-operative hip arthroscopy after femoral head OATS. (A) Patient 1. (B) Patient 2.

### Surgical technique

The technique for femoral head OATS has been previously well-described [29, 30]. A fresh frozen age and size-matched allograft was acquired pre-operatively for all patients. In this series, all grafts were procured from a consistent tissue bank source in partnership with industry support (Arthrex Inc. in Naples, Florida; LifeNet Health in Virginia Beach, Virginia; and Joint Restoration Foundation in Centennial, Colorado). Grafts are typically available within 2–3 months, and surgical planning is based on final patient agreement and availability of all involved parties.

During the procedure, the patient is placed in a lateral decubitus position under general anaesthesia. A lateral approach facilitates exposure of the peri-trochanteric space, and a trochanteric osteotomy is performed with an oscillating saw. In cases where the patient exhibits increased soft tissue laxity and hypermobility, a trochanteric osteotomy is not required to facilitate joint exposure. Careful elevation of the abductor and iliocapsularis permits visualization of the capsule, which is cut in a Z-fashion. Once the femoral head is exposed, the ligamentum teres is cut, and the femoral head is dislocated anteriorly.

Once the femoral head is circumferentially visualized, the zone of AVN/degeneration is identified. Sizing templates are then used to delineate the extent of the lesion. A central guide pin is placed through the recipient template and advanced until it is secure within the femoral head. A scoring reamer is then placed over the guide pin and used to score the articular cartilage and prepare the surface for the coring reamer. The coring reamer is then manually advanced to a depth of 10–12 mm until a bleeding surface is noted. The depth of the reaming is based on the extent and depth of the lesion with the goal of removing all necrotic bone and establishing a favourable biologic environment for graft incorporation. Next, a specialized femoral head donor jig and harvesting station is used to create a donor plug of perfect dimensions (Arthrex Inc). This is contoured with an oscillating saw based on depth measurements of the recipient socket.

**Figure 3:**
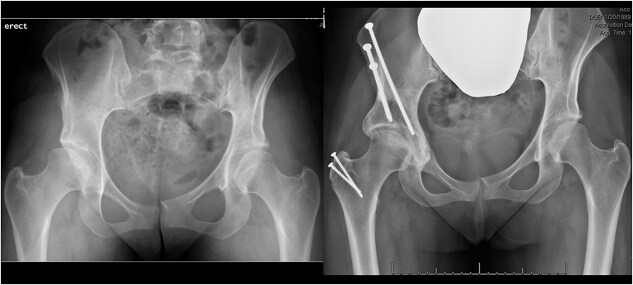
Representative pre-op and post-op radiographs of a patient who underwent combined OATS and PAO. Left: Pre-op AP pelvis. Right: One year post-op AP pelvis.

Once the graft has been appropriately prepared, it is manually placed into the recipient socket and gently tapped in place with a barrier between cartilage and mallet to protect the cellular viability of the graft [21, 28, 31]. The use of allograft offers the advantage of precise sizing, a robust and supportive graft construct, and stable press-fit fixation. If there are areas of discontinuity, additional allogenic cartilage matrix is used as a ‘grout’ to enhance the surface and graft interface [BioCartillage® Extracellular Matrix (Arthrex, Naples, Florida, USA)] (Fig. 2). At this point, the femoral head is reduced, and the hip is taken through a physiologic range of motion to determine if there is evidence of gross edge loading at the graft border or if adequate coverage and balanced force distribution are present. If it appears that the graft is being asymmetrically loaded, the decision is made to proceed with a PAO using established techniques [32]. PAO is used in these cases with severe dysplasia in order to prevent further damage to the femoral head. This procedure improves hip alignment, reducing future complications and improving pain and function [33, 34]. The capsule is then repaired, and the greater trochanter is reduced and secured using cortical screws. If extra-articular impingement, or trochanteric-pelvic impingement, is present, as determined at the time of the dynamic assessment, a relative femoral neck lengthening and trochanteric distalization is performed (Fig. 3) [35]. This was the case for two patients in our study (Table 1). The remainder of the tissue layers are closed in a typical fashion.

**Table 1:** Demographic and clinical characteristics of patients included in the study. Patients are separated into two categories: those who underwent OATS only, and those who underwent combined OATS and PAO. Averages for all patients are included at the bottom.

Patient background								
**Patient**	**Procedure**	**Degeneration cause/risk factor**	**Femoral neck lengthening**	**Previous procedure**	**Age (years)**	**Gender**	**BMI (kg/m** ^**2**^ **)**	**Side**
1	OATS	Legg–Calvé–Perthes disease	No	PAO	18	M	30.8	R
2	OATS	Sickle cell disease	Yes	N/A	13	F	21.1	R
3	OATS	Legg–Calvé–Perthes disease	Yes	Acetabular osteotomy	23	F	19.7	R
4	OATS	Long-term steroids (polyarteritis nodosa)	No	N/A	18	F	26.4	R
5	OATS	Long-term steroids (ulcerative colitis)	No	Core decompression	25	F	33.3	L
**Average**					19.4 ± 4.7	80% F	26.3 ± 5.9	80% R
**Patient**	**Procedure**	**Degeneration cause/risk factor**	**Femoral neck lengthening**	**Previous procedure**	**Age (years)**	**Gender**	**BMI (kg/m** ^**2**^ **)**	**Side**
6	OATS+PAO	Hip trauma	No	N/A	23	F	25.6	R
7	OATS+PAO	Idiopathic	No	Pelvic dega osteotomy	17	M	23.6	L
8	OATS+PAO	Sickle cell disease	No	N/A	12	F	27.5	R
9	OATS+PAO	Idiopathic	No	N/A	23	F	33.4	R
10	OATS+PAO	Legg–Calvé–Perthes disease	No	N/A	17	M	23.3	R
11	OATS+PAO	Idiopathic	No	N/A	14	F	38	R
**Average**					17.7 ± 4.5	67% F	28.6 ± 5.9	85% R
		**Age (years)**	**Gender**	**BMI (kg/m** ^**2**^ **)**			**Side**	
**Average for all patients**		**18.5 ± 4.5**	**73% F**	**27.5 ± 5.7**			**82% R**	

Patients are separated into two categories: those who underwent OATS only, and those who underwent combined OATS and PAO. Averages for all patients are included at the bottom.

**Table 2:** Demographic and clinical characteristics of patients included in the study. Patients are separated into two categories: those who underwent OATS only, and those who underwent combined OATS and PAO. Averages for all patients are included at the bottom.

Postoperative recovery							
**Patient**	**Procedure**	**Weight bearing (weeks)**	**Return to baseline physical activity (months)**	**Baseline physical activity**	**Time to THA (years)**		**Follow up (years)**
1	OATS	4	7	Manual labour	N/A		3.22
2	OATS	12	N/A	N/A	N/A		6.74
3	OATS	3	12	Swimming	N/A		9.14
4	OATS	8	N/A	N/A	N/A		6.59
5	OATS	5	5	Long walks	N/A		7.22
**Average**		6.4 ± 3.6	8.0 ± 3.6				6.5 ± 2.1
**Patient**	**Procedure**	**Weight bearing (weeks)**	**Return to baseline physical activity (months)**	**Baseline physical activity**	**Time to THA (years)**		**Follow up (years)**
6	OATS+PAO	8	9	Recreational sports	N/A		10.98
7	OATS+PAO	20	N/A	N/A	N/A		3.14
8	OATS+PAO	16	N/A	N/A	N/A		4.02
9	OATS+PAO	16	12	Running	6		8.6
10	OATS+PAO	10	12	Baseball	N/A		1.02
11	OATS+PAO	8	6	Running	N/A		10.52
**Average**		13.0 ± 5.0	9.8 ± 2.9				6.4 ± 4.2
	**Weight bearing (weeks)**		**Return to baseline physical activity (months)**		**Time to THA (years)**		**Follow up (years)**
**Average for all patients**	10.0 ± 5.5		9.0 ± 3.1		6.0		6.5 ± 3.3

**Table 3:** Demographic and clinical characteristics of patients included in the study. Patients are separated into two categories: those who underwent OATS only, and those who underwent combined OATS and PAO. Averages for all patients are included at the bottom.

Acetabular coverage						
**Patient**	**Procedure**	**Preop LCEA (degrees)**	**Preop acetabular index (degrees)**	**Postop LCEA (degrees)**	**Postop acetabular index (degrees)**	**Follow up (years)**
1	OATS	38.1	0.6	36.5	0.6	3.22
2	OATS	19.3	5.6	16.5	5.6	6.74
3	OATS	22.5	4.3	23.7	6.3	9.14
4	OATS	23	3.8	21	3.4	6.59
5	OATS	25.7	10	25.6	9.6	7.22
**Average**		25.7 ± 6.5	4.9 ± 3.1	24.7 ± 6.7	5.1 ± 3.0	6.5 ± 2.1
**Patient**	**Procedure**	**Preop LCEA (degrees)**	**Preop acetabular index (degrees)**	**Postop LCEA (degrees)**	**Postop acetabular index (degrees)**	**Follow up (years)**
6	OATS+PAO	11	18.1	29.4	1.6	10.98
7	OATS+PAO	15.8	13.2	26.3	8.4	3.14
8	OATS+PAO	15.9	4.6	23.1	3.2	4.02
9	OATS+PAO	11.8	12.2	20.9	3.1	8.6
10	OATS+PAO	17.8	13.2	25	7.5	1.02
11	OATS+PAO	6.5	27.2	30.6	8.5	10.52
**Average**		13.1 ± 3.8	14.8 ± 6.8	25.9 ± 3.4	5.4 ± 2.8	6.4 ± 4.2
	**Preop LCEA (degrees)**	**Preop acetabular index (degrees)**	**Postop LCEA (degrees)**	**Postop acetabular index (degrees)**	**Follow up (years)**	
**Average for all patients**	18.9 ± 8.2	10.3 ± 7.3	25.3 ± 5.2	5.3 ± 2.9	6.5 ± 3.3	

**Table 4:** Pre-operative and post-operative range of motion.

**OATS-only range of motion**							
**Patient**	**Procedure**	**Preoperative**			**Postoperative**		
		**Flexion**	**IR**	**ER**	**Flexion**	**IR**	**ER**
1	OATS	120	10	30	110	20	40
2	OATS	90	40	40	100	5	40
3	OATS	80	5	30	120	20	50
4	OATS	100	10	15	120	20	60
5	OATS	90	10	70	100	20	60
**Mean**		95	17.5	40.8	111.7	17.5	53.3
**OATS + PAO range of motion**							
**Patient**	**Procedure**	**Preoperative**			**Postoperative**		
		**Flexion**	**IR**	**ER**	**Flexion**	**IR**	**ER**
6	OATS+PAO	90	50	60	120	30	30
7	OATS+PAO	100	28	34	110	30	45
8	OATS+PAO	80	10	10	110	10	40
9	OATS+PAO	90	40	45	110	30	45
10	OATS+PAO	100	0	60	120	20	60
11	OATS+PAO	90	30	60	120	20	70
**Mean**		91.7	26.3	44.8	115	23.3	48.3
**All patients’ range of motion**							
		**Preoperative**			**Postoperative**		
		**Flexion**	**IR**	**ER**	**Flexion**	**IR**	**ER**
Mean		93.6	21.2	41.3	112.7	20.5	49.1
SD		11.2	17.0	19.7	7.9	7.9	12.0
*P*-value					0.001	0.887	0.223

IR, internal rotation. ER, external rotation. SD, standard deviation.

### Statistical analysis

The statistical analysis utilized SPSS 29.0 software (IBM Corporation, Endicott, NY) and focused on PROMIS domains. Averages and standard deviations (SD) were computed for these domains. Fisher’s exact analysis was employed to evaluate significant differences in categorical data between individuals who underwent a PAO and those who did not receive a concurrent PAO with the OATS procedure. Additionally, bivariate Student’s *t*-test analysis was conducted to assess differences in continuous variables between the two groups, because the data were found to follow a normal distribution. Statistical significance was set at a *P*-value < .05.

## RESULTS

Of the 11 patients in the study, eight were female and three were male. The average age was 19.9 ± 5.4 years, and the average BMI was 26.1 ± 4.7 kg/m^2^. Risk factors for femoral head degeneration included Legg-Calvé-Perthes disease, sickle cell disease, previous hip trauma, and long-term steroid use (Table 1). All patients demonstrated radiographic evidence of focal degeneration of the femoral head based on MRI and X-ray findings (Appendix 1). One patient had bone marrow oedema (BME) surrounding their AVN and was preoperatively treated with parathyroid hormone for 3 months to allow consolidation of the BME. The average fresh femoral head allograft size was 25.0 ± 3.5 mm. Four patients had prior procedures involving their surgical hip (Table 1).

Average follow-up for all patients included in the study was 6.5 ± 3.3 years. Postoperatively, patients were able to transition to full weight bearing by an average of 9.8 ± 5.6 weeks. Seven patients were able to return to sports or baseline physical activities at an average interval of 9.9 ± 2.9 months. Baseline physical activities included running, baseball, swimming, manual labour, and other recreational sports. Two patients did not return to sports during the OATS recovery period due to subsequent knee surgery. One patient did not return to sports during the OATS recovery period due to surgery on the contralateral hip. One patient did not return to sports because of unrelated chronic health conditions (Table 2). All patients started rehabilitation exercises within a few days of surgery with inpatient physical therapy, and they all attended outpatient physical therapy within 2–3 weeks of hospital discharge.

With respect to range of motion, there was a significant increase in average hip flexion from 93.6 ± 11.2° to 112.7 ± 7.9° (*P* = .001) (95% CI: 10.98°, 27.22°). There was no significant change in internal rotation from pre-op to post-op (21.2 ± 17.0° and 20.5 ± 7.9°, respectively, *P* = .89) (95% CI: −11.77°, 10.37°). There was also no significant change in external rotation from pre-op to post-op (41.3 ± 19.7° and 49.1 ± 12.0°, respectively, *P* = .22) (95% CI: −5.83°, 21.43°). There was no difference in post-operative range of motion with respect to flexion, IR, or ER between patients who received OATS and OATS/PAO (all *P*-values > .05) (Table 4). One patient in the study cohort had undergone conversion to THA at the final follow-up, six years from the time of their original OATS procedure. The average follow-up of the remaining patients was 6.5 ± 3.3 years (Table 2).

Six patients included in the study cohort underwent simultaneous PAO at the time of their femoral head OATS procedure. The average age of these patients was 20.3 ± 6.3 years. Four were female and two were male. The average follow-up among these patients was 6.4 ± 4.2 years. Weight bearing transition in combined OATS/PAO patients took an average of 12.7 ± 5.5 weeks to return to full weight bearing status, whereas isolated OATS patients were able to transition to full weight bearing by an average of 6.4 ± 3.6 weeks (Table 2). Six patients had frank ipsilateral hip dysplasia, defined as an LCEA below 18°, and three patients had ipsilateral borderline hip dysplasia, defined as an LCEA between 19 and 24° [36]. The average preoperative LCEA for patients who underwent both OATS and PAO was 13.1 ± 3.8°, and their average acetabular index was 14.8 ± 6.8°. The average postoperative LCEA for patients who underwent both OATS and PAO was 25.9 ± 3.4°, and their average acetabular index was 5.4 ± 2.8°. For the patients who underwent an isolated OATS procedure, the average preoperative LCEA was 25.7 ± 6.5° and the average acetabular index was 14.9 ± 3.1°. These patients’ average postoperative LCEA was 24.7 ± 6.7° and their average acetabular index was 5.1 ± 3.0° (Table 3).

**Figure 4:**
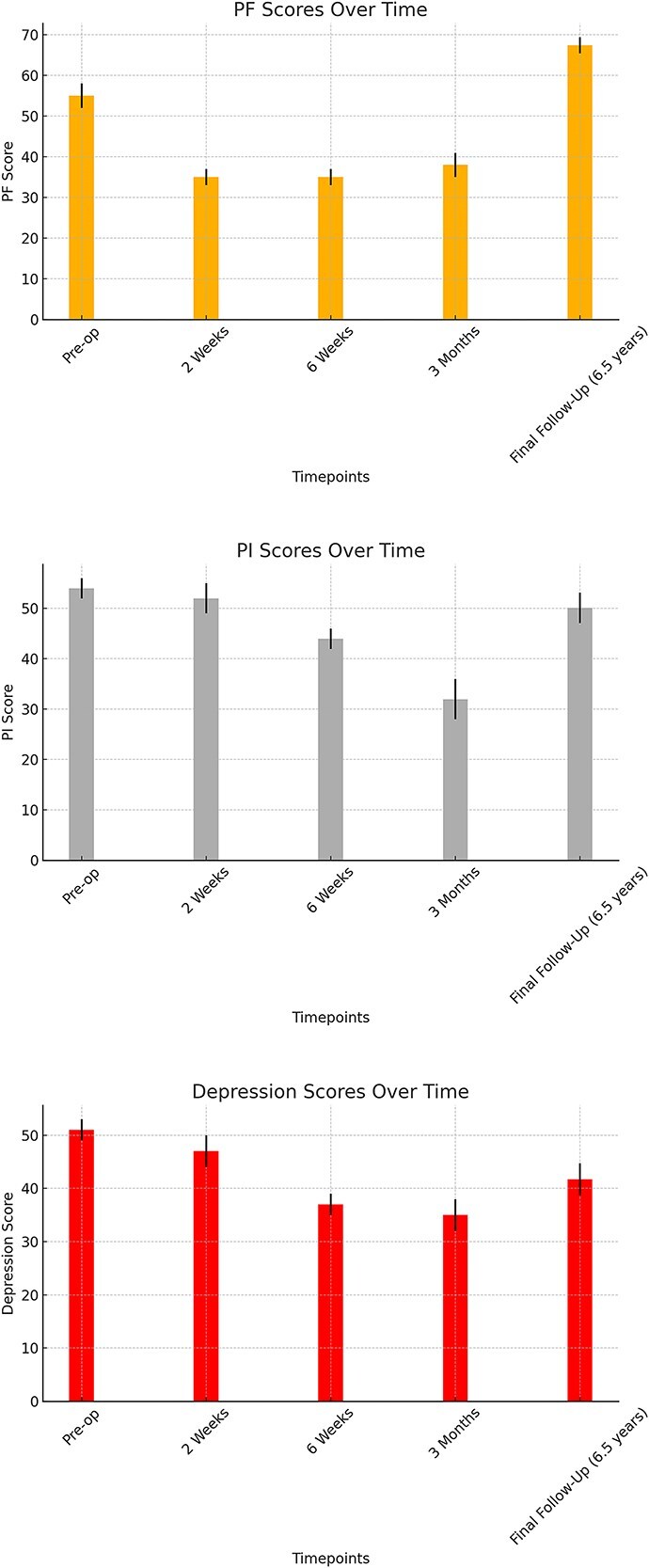
PROMIS outcomes. PROMIS PF, PI, and D scores for preoperative, 2-week, 6-week, and final follow-up timepoints. Final follow-up scores are from an average of 6.5 years after surgery. Error bars represent the standard error of each mean.

Five patients were able to be reached for telephone follow-up. Four were pleased with their results, and one patient was in continued pain and displeased with their current level of function. The PROMIS analysis demonstrated a significant average improvement in PF scores at the final telephone follow-up compared to the preoperative scores within the entire cohort (*P* = .01) (95% CI: 10.02, 35.98). Patients originally had significantly worse PF scores at 2 weeks, 6 weeks, and 3 months postoperatively, but by the time of final follow-up, the average PF score was significantly improved from the preoperative score. In terms of PI and depression, there was no significant change seen at the final follow-up compared with preoperative scores (*P* = .31) (95% CI: −24.25,4.25) and (95% CI: −17.99,7.99), respectively (Fig. 4).

## DISCUSSION

### Summary of findings

Here, we report favourable results of 11 femoral head OATS procedures performed for focal degeneration with subchondral collapse of the femoral head. All patients returned to weight bearing and sports or baseline physical activity within acceptable timeframes for their procedures, and the four patients who did not return to sports were hindered by health conditions unrelated to their surgical procedure. Patients’ postoperative hip flexion increased significantly from 93.6 ± 11.2° to 112.7 ± 7.9° (*P* = .001) (95% CI: 10.98°, 27.22°). The American Academy of Orthopaedic Surgeons defines the normal range of hip flexion as 128.4 ± 6.7° [37], which suggests that the OATS procedure brought these patients’ hip flexion closer to normal range. In terms of hip dysplasia, OATS and PAO combined patients had a lower mean preoperative LCEA of 13.1 ± 3.8°, and a higher mean preoperative acetabular index of 14.8 ± 6.8°. The patients who underwent isolated OATS alone demonstrated a more physiologic mean preoperative LCEA of 25.7 ± 6.5° (*P* = .02), and a more physiologic acetabular index of 4.9 ± 3.1° (*P* < .5). The average LCEA and acetabular indices of OATS-only patients remained physiologic preoperatively and postoperatively, while these values for OATS and PAO patients improved postoperatively. The average postoperative LCEA for OATS and PAO patients was in the physiologic range at 25.9 ± 3.4°. Similarly, the average postoperative acetabular index for these patients was physiologic at 5.4 ± 2.8°. Four of the five contacted patients expressed they were pleased with their hip function and pain levels, and one patient was experiencing persistent pain at the time of final follow-up (4 years postop). One patient progressed to THA, and the remaining patients were able to retain their native hips (survivorship of 91% at an average of 6.5 ± 3.3 years postop).

### Comparison with prior studies

Overall, other studies also suggest that allogenic bone grafting, *via* OATS or another method, is an effective treatment for focal degeneration of the femoral head with subchondral collapse. Two case series demonstrated favourable results of OATS: one included 22 patients [28], and the other included eight patients [21]. Graft survivorship in the larger study was reported at 78.5% at 5 years [28], in the smaller study was 86% at 2 years and 67% at 9 years [21], and in our study was 91%, at an average of 6.5 ± 3.3 years. The present study is unique in that it is the only series to include patients who underwent simultaneous femoral head OATS and PAO. Outside of these case series, there is a recent case report describing OATS as a successful treatment for an individual with an osteochondral defect of the femoral head [38].

### Implications and clinical considerations

We found that a considerable number of patients who displayed radiographic evidence of focal femoral head degeneration also demonstrated acetabular dysplasia, as measured by LCEA and acetabular index. The single patient in our series who was eventually converted to THA demonstrated the most severe acetabular dysplasia among the group, with an LCEA of 12.4° and an acetabular index of 23.9°. For all patients, intra-operative dynamic assessment and preoperative radiographic metrics were used to determine whether PAO was necessary; six patients had a PAO procedure during their index OATS procedure. The patient who progressed to THA underwent simultaneous OATS and PAO, while none of the patients who exclusively underwent OATS went on to have a THA. Four patients in total underwent other secondary procedures following their OATS. These included arthroscopic intervention to address recurrent synovitis, capsulolabral adhesions, persistent anatomic conflict, and symptomatic labral pathology. Intraoperative findings in both cases demonstrated excellent graft incorporation with a nearly seamless transition of host chondral surface to graft interface (Fig. 2).

### Limitations and future directions

There are several limitations in this study. With only 11 patients, our sample size is small, and this limits the generalizability of our findings. Further, we were only able to reach five patients to gather updated PROMIS data, so many of our conclusions are based on retrospective chart review rather than the PROMIS data. When comparing preoperative and postoperative PROMIS scores, we also need to keep in mind that many of the patients went on to have other procedures after their OATS procedure, which tend to have an impact on PROMIS scores. It is worth noting that the PROMIS tool was originally validated for computer rather than telephone administration, so although it is widely administered in both formats, it is possible that telephone administration could skew the results. Lastly, although we have preoperative MRI studies, most patients do not have a postoperative MRI since these are not routinely performed in asymptomatic patients. The best supports we have for graft incorporation are the intraoperative images seen in Fig. 2.

## CONCLUSION

Focal femoral head degeneration with subchondral collapse can result in debilitating pain and loss of mobility for patients, and a consistently successful treatment has yet to be identified. The femoral head OATS procedure has become more popular in recent years and may be a reliable treatment for this problem. Patients in our series demonstrated sustained favourable outcomes for functional gain at the time of their final follow-up. The femoral head OATS procedure may be a reliable treatment with potential to delay THA. In patients with concomitant dysplasia, combined OATS and PAO may provide further enhancement and durability of surgical outcomes.

## Supplementary Material

Appendix_Preoperative_and_Postoperative_Imaging_Data_(2)_hnaf037

## Data Availability

Any data not incorporated into this article is available upon request.
