# Supplementary material for: Femoral head osteochondral allograft transplantation with and without simultaneous periacetabular osteotomy: a case series
Source: J Hip Preserv Surg. 2025 Aug 4;13(1):26–34. doi: 10.1093/jhps/hnaf037 (PMC12891996; doi:10.1093/jhps/hnaf037)
Supplement: Appendix_Preoperative_and_Postoperative_Imaging_Data_(2)_hnaf037 [file appendix_preoperative_and_postoperative_imaging_data_(2)_hnaf037.docx]

Appendix: Preoperative and Postoperative Imaging

Patient 1: Steinberg Classification III

**Preoperative MRI:**


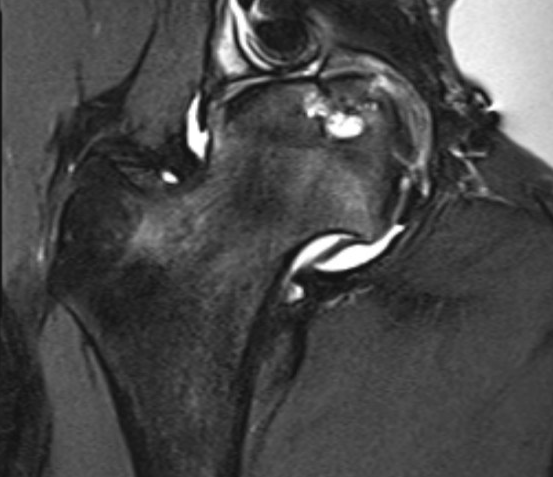


**Preoperative X-ray:**

**
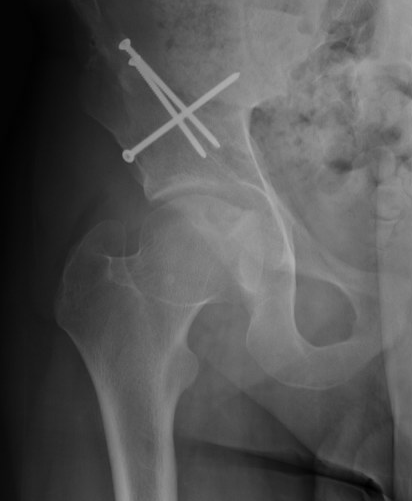
**

**Postoperative X-ray:**

**
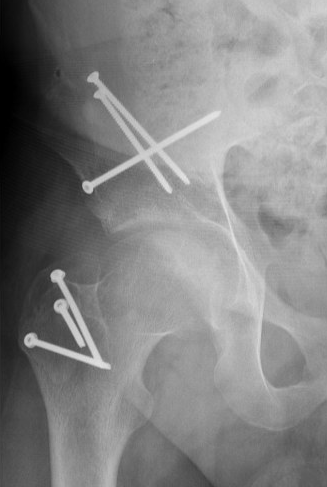
**

Patient 2: Steinberg Classification IV

**
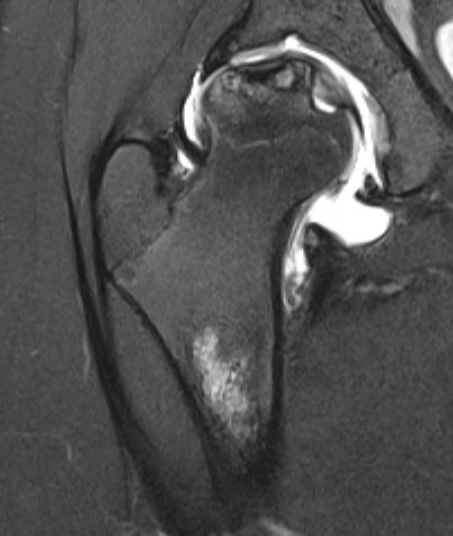
Preoperative MRI:**

**
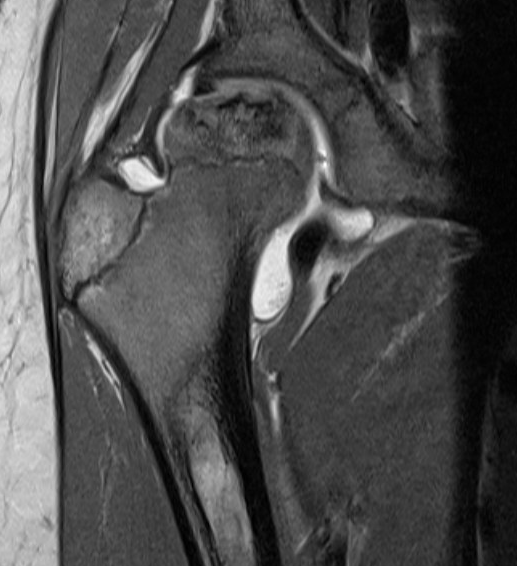
** **Preoperative X-ray:**

**
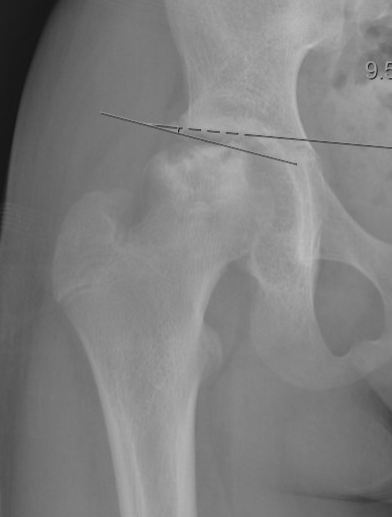
**


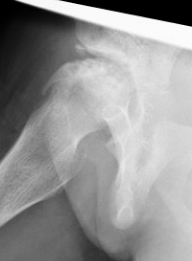


**Postoperative X-ray:**


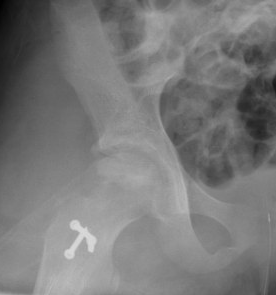


Patient 3: Steinberg Classification II

**Preoperative CT:**

**
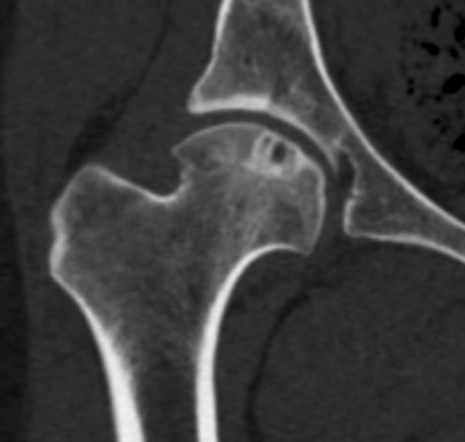
**

**Preoperative X-ray:**

**
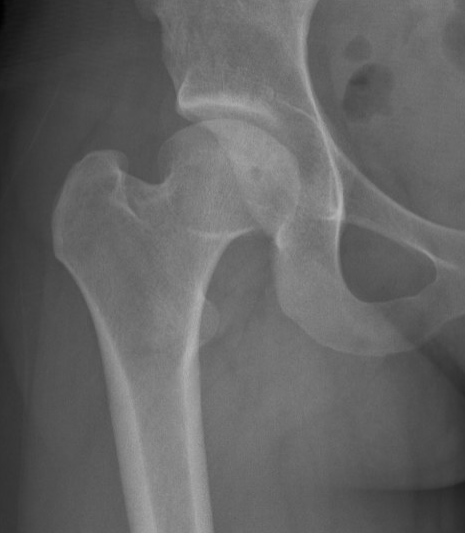
**

**
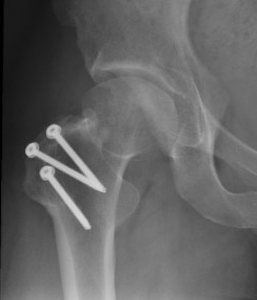
Postoperative X-ray: Postoperative MRI:**


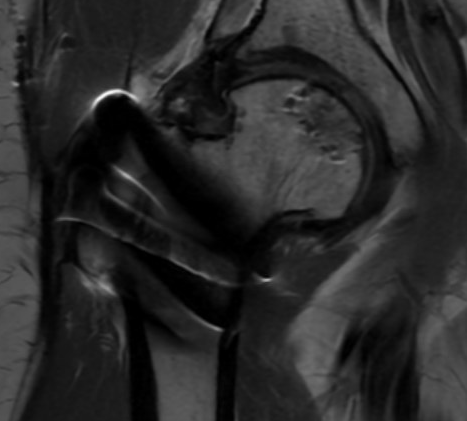


Patient 4: Steinberg Classification II


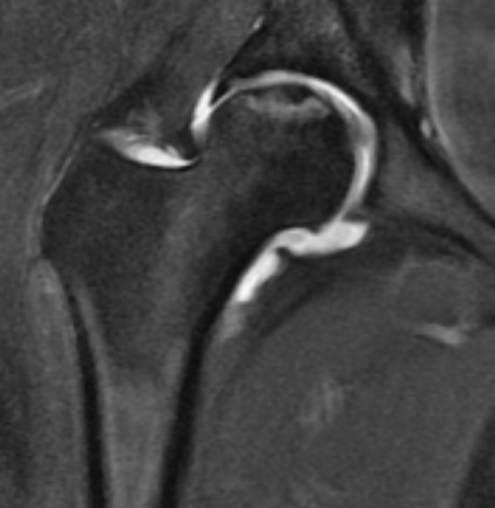

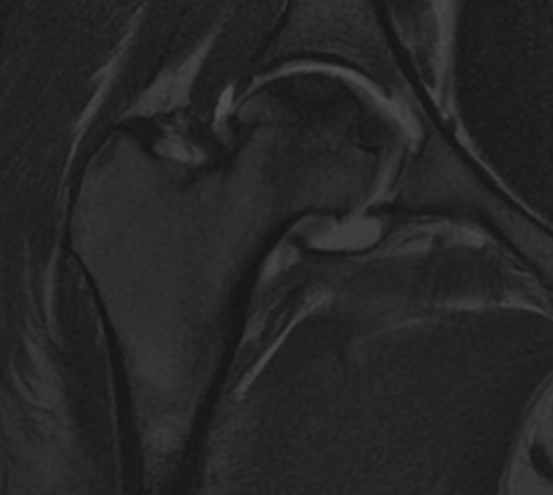
**Preoperative MRI:**

**Preoperative X-ray:**

**
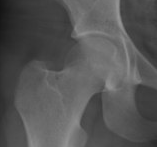
**

**Postoperative X-ray:**

**
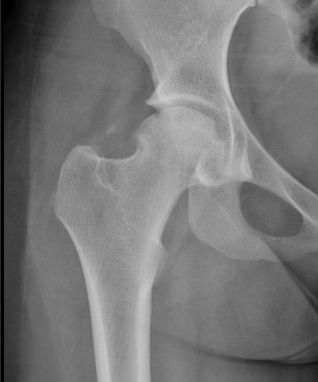
**

Patient 5: Steinberg Classification III


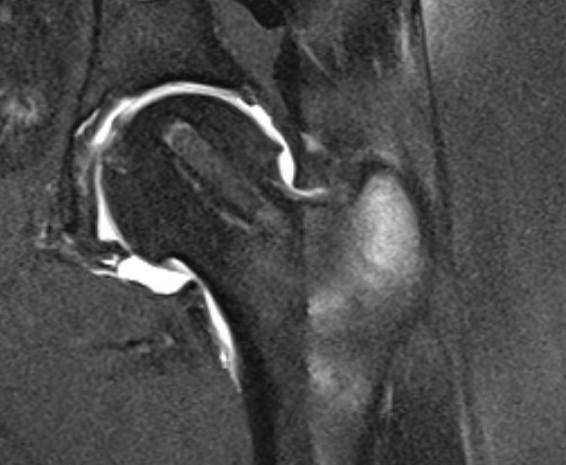

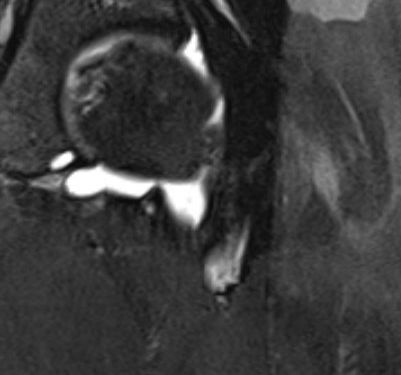
**Preoperative MRI:**

**Preoperative x-Ray:**

**
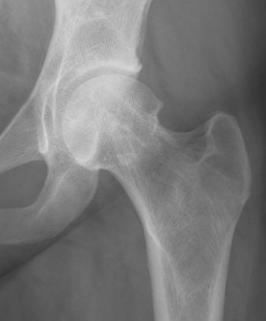
**

**Postoperative X-ray:**

**
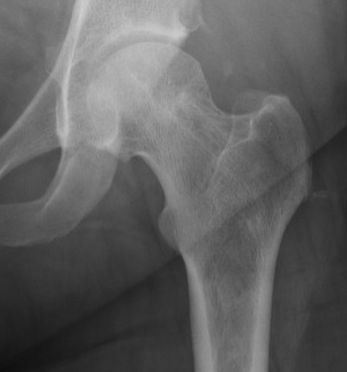
**

**Postoperative MRI:**


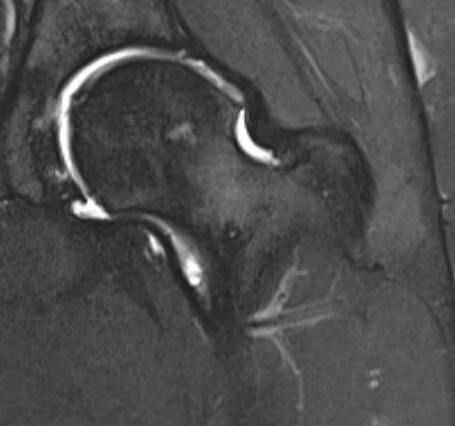

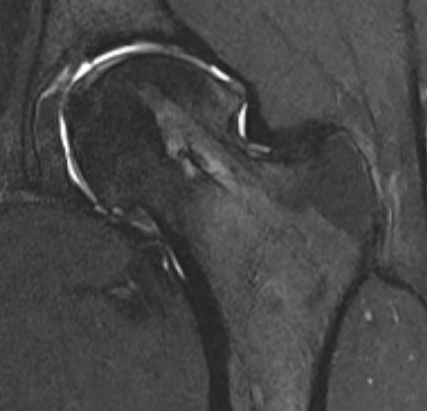


Patient 6: Steinberg Classification II

**Preoperative X-ray:**

**
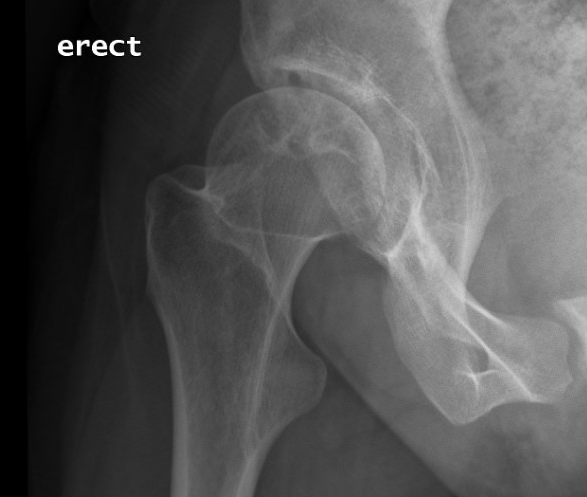
**

**Postoperative X-ray:**

**
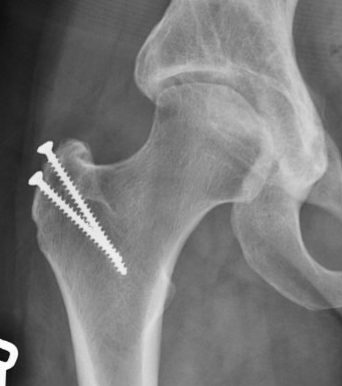
**

Patient 7: Steinberg Classification V

**
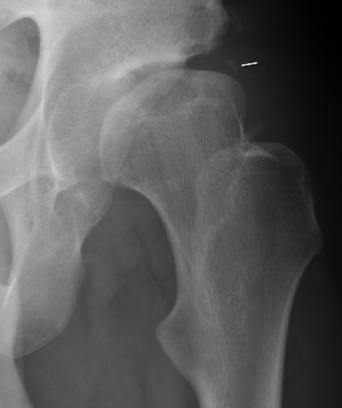

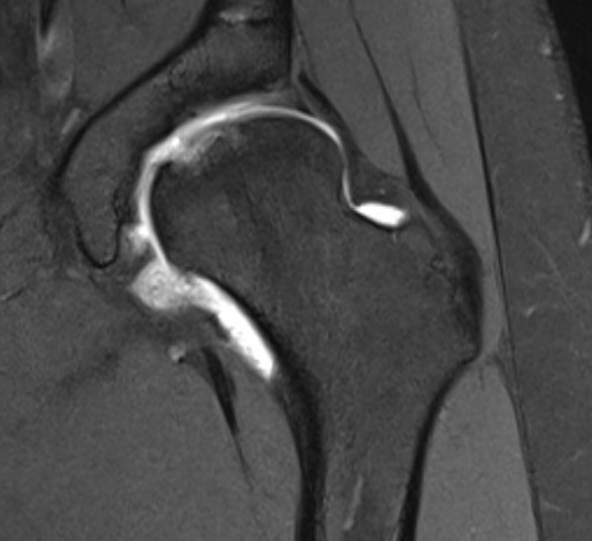
Preoperative MRI: Preoperative X-ray:**

**Postoperative X-ray:**

**
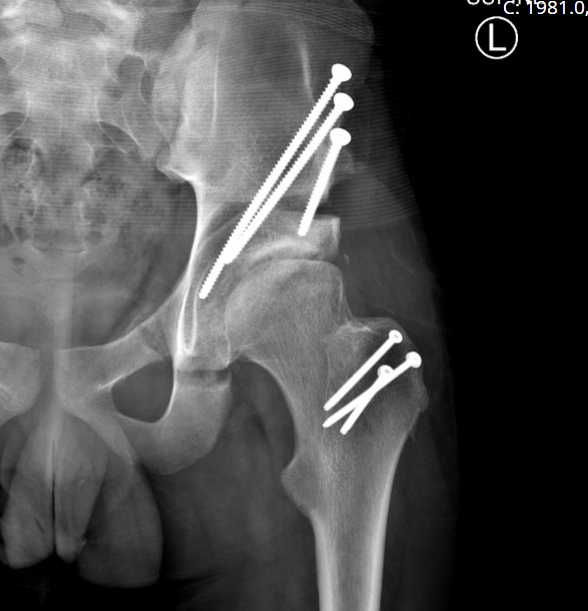
**

Patient 8: Steinberg Classification V

**Preoperative MRI:**

**
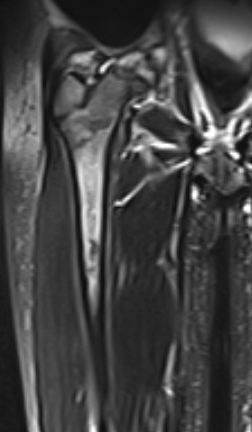
**

**Preoperative X-ray:**

**
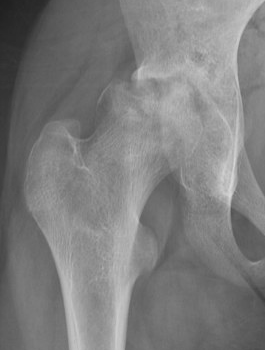
**

**Postoperative X-ray:**


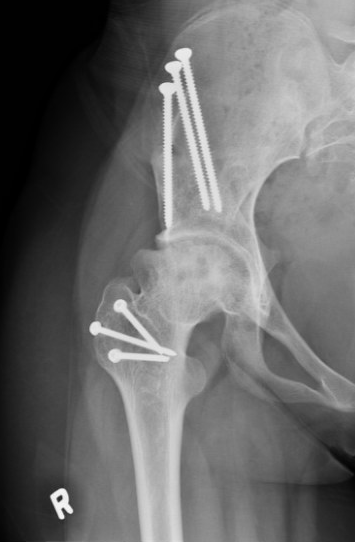


Patient 9: Steinberg Classification III


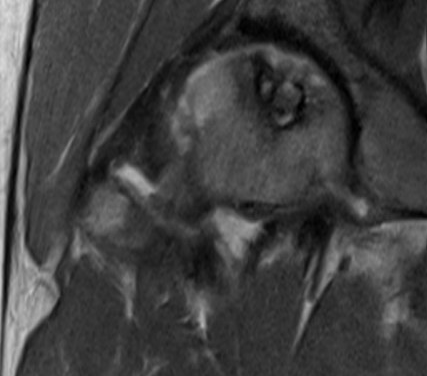
**
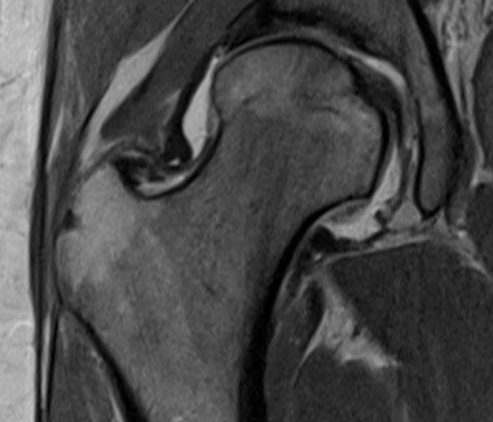
Preoperative MRI:**

**Preoperative X-ray:**

**
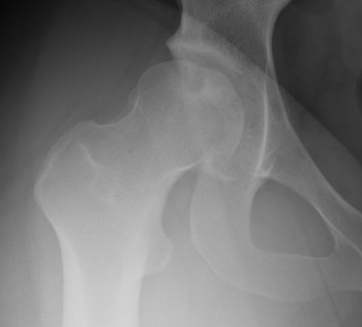
**

**Postoperative X-ray:**

**
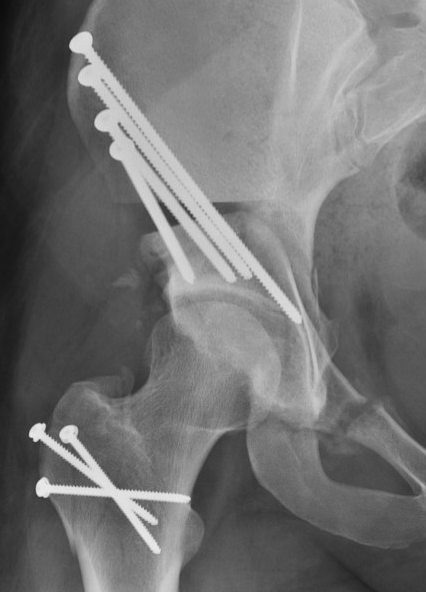
**


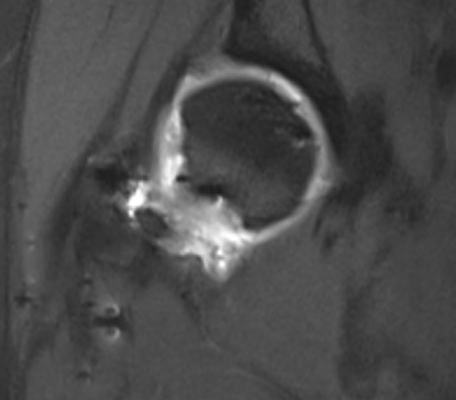
**
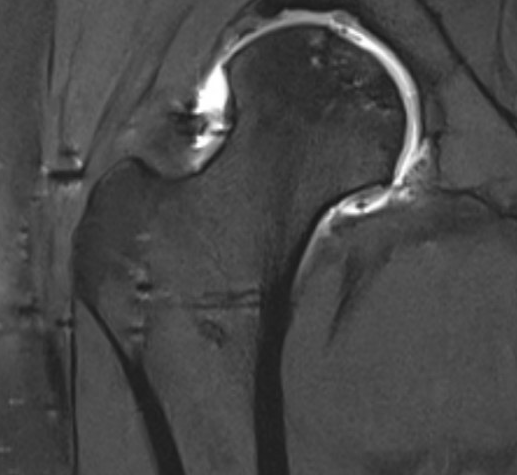
Postoperative MRI:**

Patient 10: Steinberg Classification IV

**Preoperative MRI:**

**
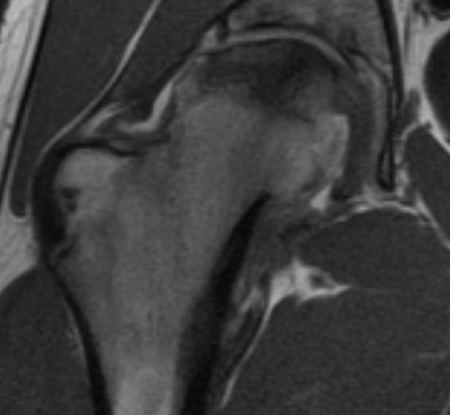
**

**Preoperative X-ray:**

**
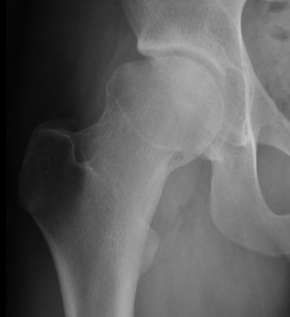
**

**Postoperative X-ray:**

**
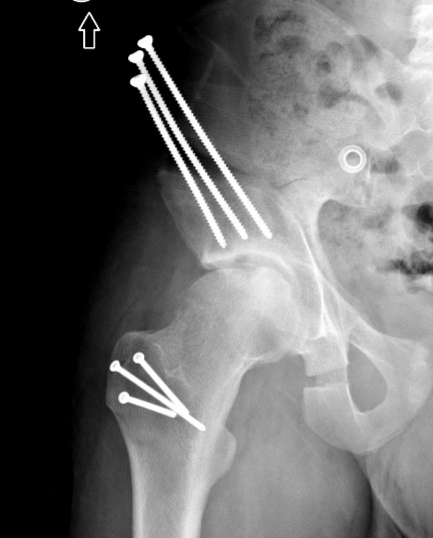
**

Patient 11: Steinberg Classification III

**Preoperative CT:**

**
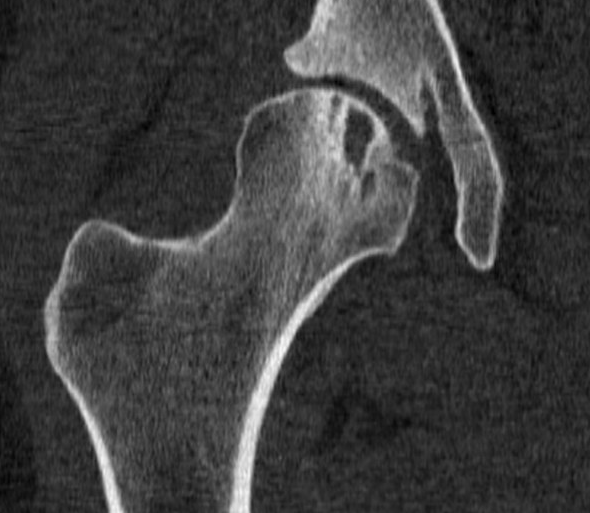
**

**Preoperative X-ray:**

**
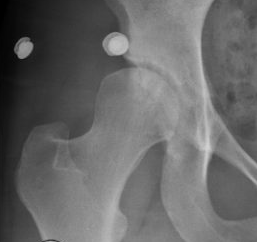
**

**Postoperative X-ray:**

**
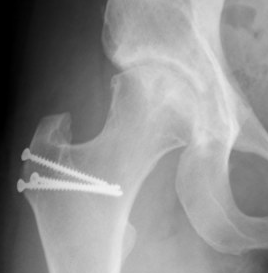
**
